# Supplementary material for: A Homeostasis Hypothesis of Avian Influenza Resistance in Chickens
Source: Genes (Basel). 2019 Jul 17;10(7):543. doi: 10.3390/genes10070543 (PMC6678902; doi:10.3390/genes10070543)
Supplement: Supplementary file 1 [file genes-10-00543-s001.pdf]

genes

MDPI

Supplementary Materials

A Homeostasis Hypothesis of Avian Influenza Resistance in Chickens

Jing An [1,2], Jinxiu Li [2,†], Ying Wang [3], Jing Wang [2], Qinghe Li [2], Huaijun Zhou [3], Xiaoxiang Hu [2,†,*], Yiqiang Zhao [1,2,†,*] and Ning Li [2]

1 Beijing Advanced Innovation Center for Food Nutrition and Human Health, College of Biological Sciences, China Agricultural University, Beijing 100193, China
2 State Key Laboratory of Agrobiotechnology, College of Biological Sciences, China Agricultural University, Beijing 100193, China
3 Department of Animal Science, University of California, Davis, California 95616, U.S.A.
† These authors contributed equally to this work
* Correspondence:huxx@cau.edu.cn (X.H.); yiqiangz@cau.edu.cn (Y.Z.)

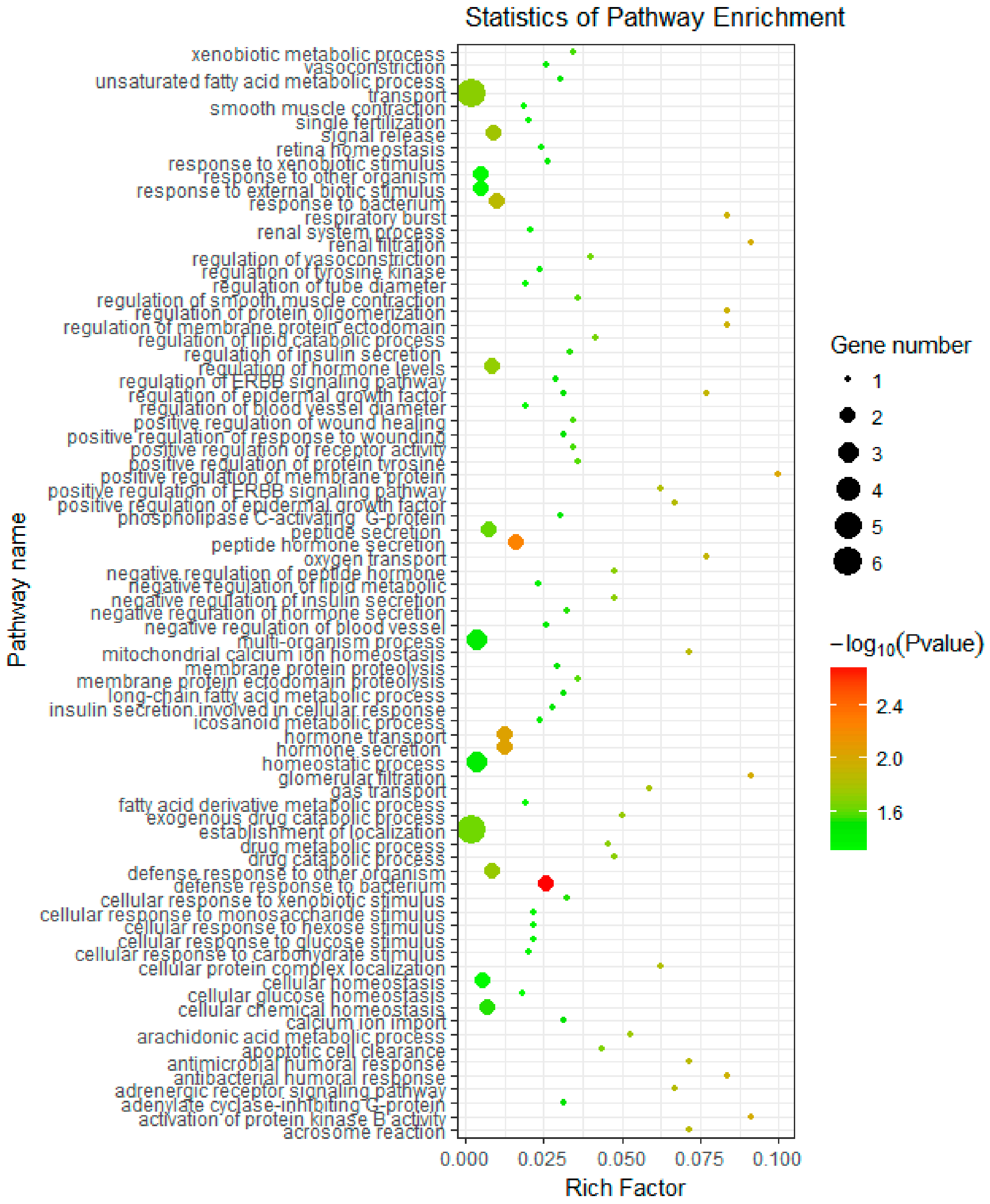

Figure S1. Pathway annotation of differentially expressed genes with FDR-corrected $p < 0.05$ for Fayoumi with avian influenza virus (AIV) inoculation compared to Fayoumi challenged with PBS.

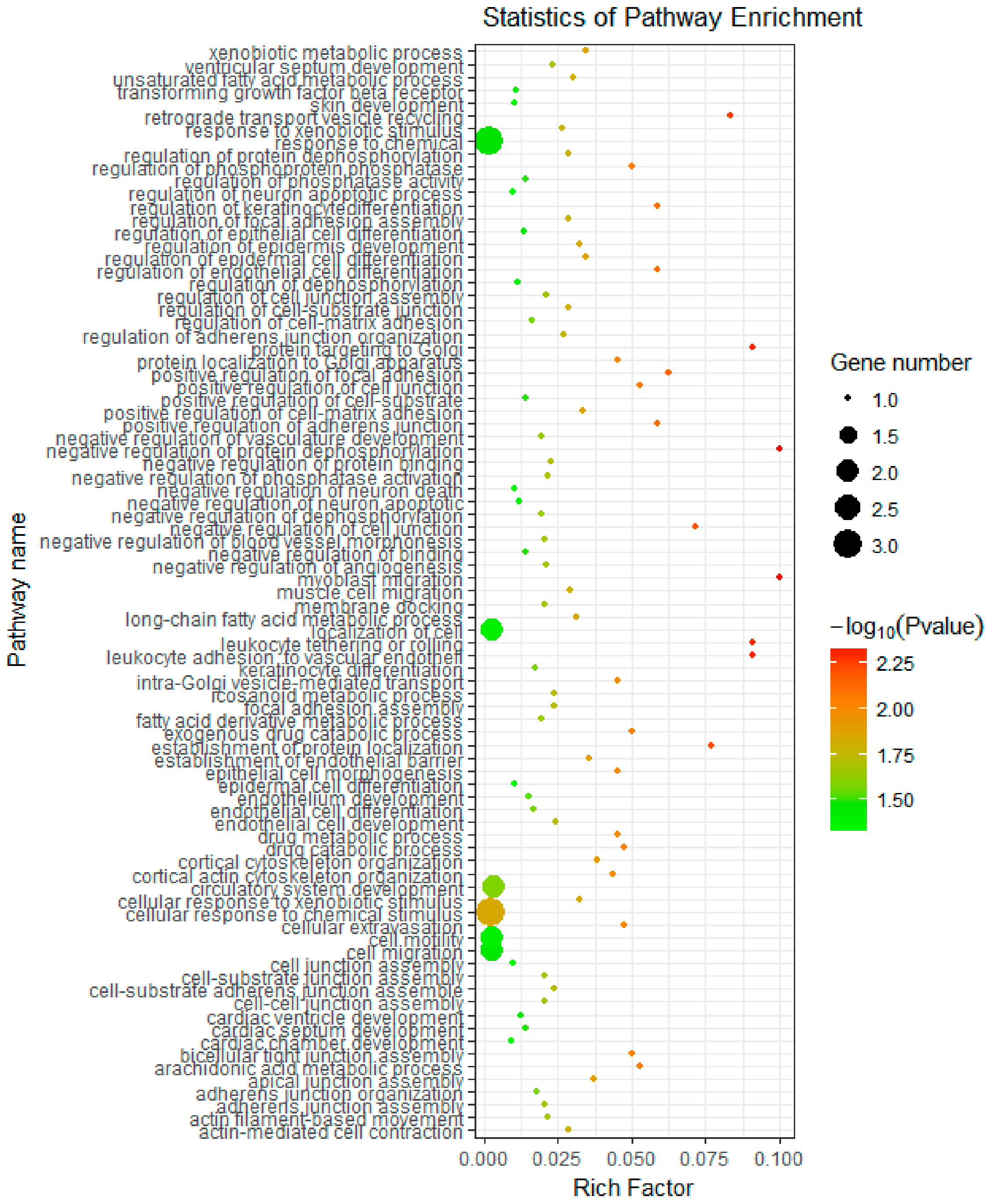

Figure S2. Pathway annotation of differentially expressed genes with FDR-corrected p < 0.05 for Leghorn chickens with AIV inoculation compared to Leghorn chickens challenged with PBS.

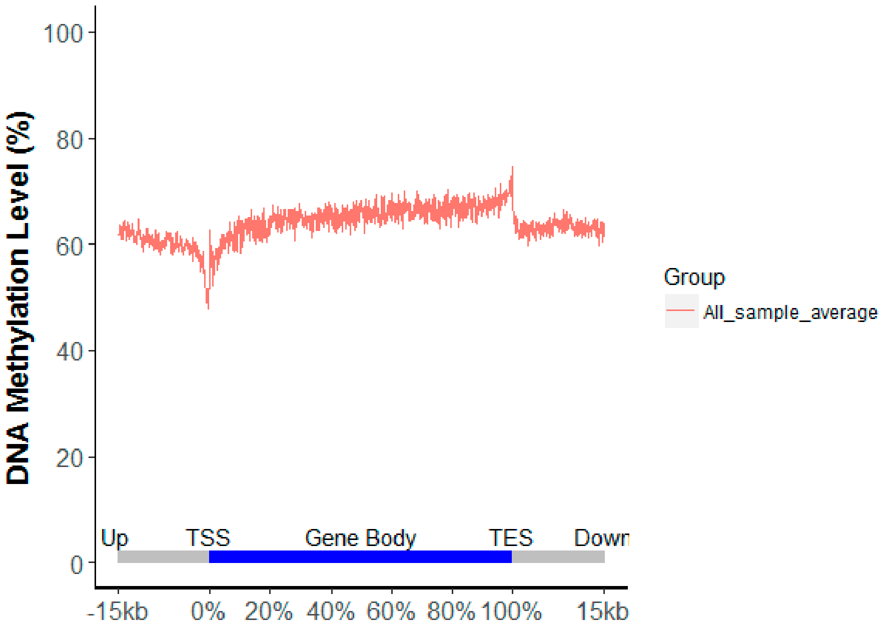

Figure S3. DNA methylation level along the gene structure in all samples.

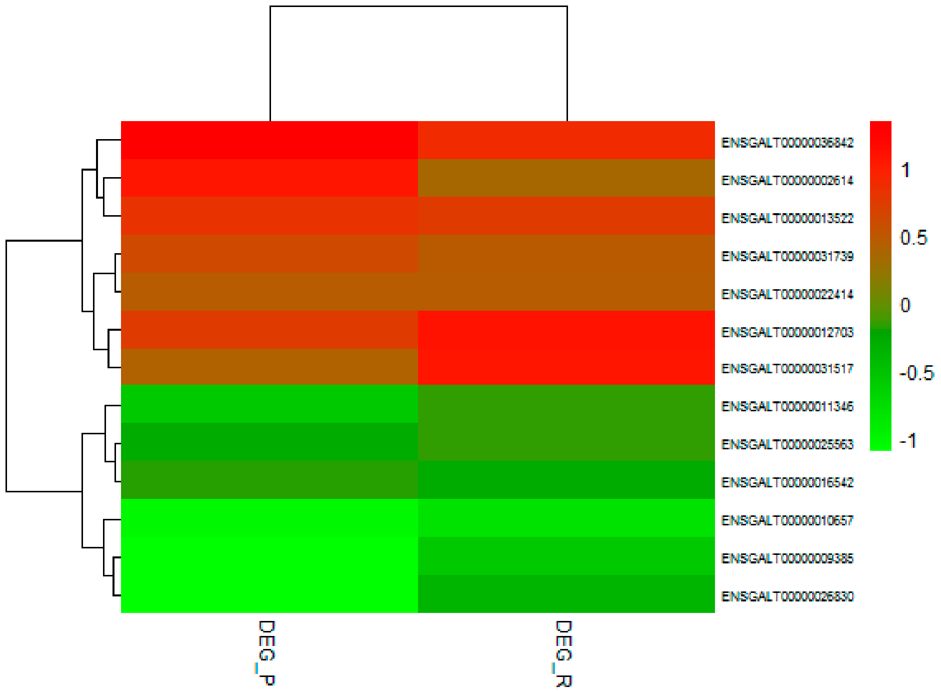

Figure S4. Heatmap of top-fold change genes with FDR-corrected $p< 0.05$ at the RNA and protein levels. "DEG_P" stands for "changes in protein level for top fold change genes with FDR-corrected P< 0.05", and the value is $\log_2$(protein expression values of individuals with AIV inoculation/protein expression values of individuals challenged with PBS). "DEG_R" stands for "changes in RNA level for top fold change genes with FDR-corrected P< 0.05", and the value is $\log_2$(gene expression values of individuals with AIV inoculation/gene expression values of individuals challenged with PBS). Same genes were compared for each line. To better demonstrate the trends, all data shown in the figure were transformed into a range from -1 to 1.

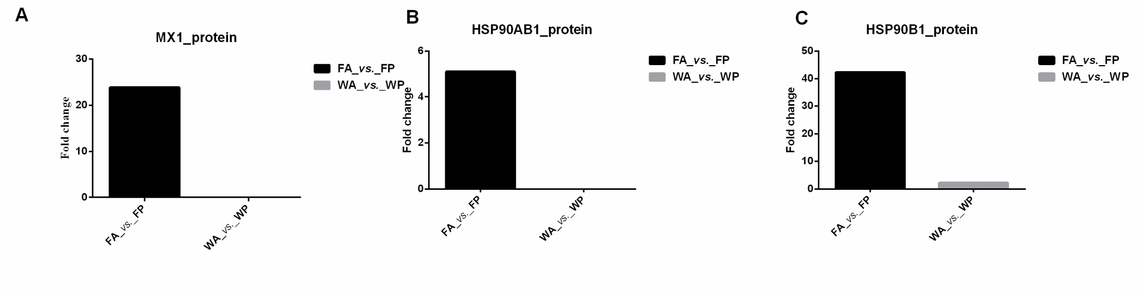

Figure S5. Protein regulation trends of *MX1, HSP90AB1, HSP90B1* between Fayoumi and Leghorn breeds. Figure A,B,C represented individual protein expression regulation trends, which were measured as log_2(protein expression values of individuals with AIV inoculation/protein expression values of individuals challenged with PBS).

Table S1. Gene expression difference and fold change levels in immune response related genes.

Name	FA *vs.* FP	LA *vs.* LP	FA *vs.* FP/ LA *vs.* LP
MX1	-0.6350	-0.0485	13.0944
NOX2	-0.7980	-0.0829	9.6298
HSP90AB1	2.8852	0.4741	6.0851
IL1B	-0.8836	-0.1606	5.5009
IL13RA1	-0.5136	-0.1082	4.7490
IL13RA2	-0.2892	-0.1167	2.4792
TLR4	-0.7811	-0.6105	1.2796
IL4I1	0.5411	0.4341	1.2466
JCHAIN	-1.7559	-1.4536	1.2080
ENSGALT00000041159	-9.6676	-10.1963	0.9481
FERMT2	0.1536	0.1926	0.7976
MHC_class_II_BLB1	-5.7237	-7.8717	0.7271
BLB1	0.7809	1.1470	0.6808

JAK1	-0.3579	-0.5695	0.6285
IRF9	-0.4740	-0.8837	0.5364
CD8A	6.7634	12.8365	0.5269
COQ10B	0.2644	0.8542	0.3095
IL13	-0.5726	-2.2261	0.2572
ATG5	0.2199	0.9593	0.2292
SASH3	-0.1380	-0.8907	0.1550
NOS2	0.1475	1.0242	0.1440
IRF7	-0.0050	-0.1395	0.0355
ACOT8	0.1084	-0.6135	-0.1766
TLR3	-0.4528	2.4010	-0.1886
ADAR	0.1058	-0.5483	-0.1930
HSP90B1	0.0845	-0.3041	-0.2780
IL18	-0.5229	1.2377	-0.4224
FERMT1	-0.0964	0.1799	-0.5357
TLR7	-3.8723	5.8492	-0.6620
IL2RB	0.3850	-0.3876	-0.9934
THOC1	0.2992	-0.2951	-1.0139
NCOA4	0.2335	-0.2199	-1.0617
JAK2	-0.5399	0.4222	-1.2787

CASP9	0.3693	-0.2732	-1.3517
CD4	0.2143	-0.1172	-1.8291
TNFAIP8L3	-0.8062	0.4224	-1.9088
CASP1	0.3631	-0.0873	-4.1589
GPR18	-1.1916	0.1520	-7.8387
IFNA3	-1.0232	0.0632	-16.1811
CIITA	-1.2400	0.0470	-26.3757

FA *vs.* FP stands for gene expression difference in Fayoumi breed, measured as $\log_2$(gene expression levels of Fayoumi individuals with AIV inoculation/gene expression levels of Fayoumi individuals challenged with PBS), LA *vs.* LP stands for gene expression difference in Leghorn breed, measured as $\log_2$ (gene expression levels of Leghorn individuals injected with AIV/ gene expression levels of Leghorn individuals challenged with PBS), FA *vs.* FP/ LA *vs.* LP represents a comparison of gene expression fold change levels between Fayoumi and Leghorn breeds, which have been ordered by magnitude (highest to lowest).

Table S2. Whole genome bisulfite sequencing (WGBS) data statistics summary.

Samples	Library	Reads	Mapped Reads	Unique mapped Reads	Mapping efficiency
Fayoumi control replicate 1	A	175421846	157879661	155774600	88.8%
	B	175900866	152506051	148900815	84.7%
Fayoumi control replicate 2	A	172418670	164659830	161215246	93.5%
	B	170400106	163413702	160061344	93.9%
	All	694141488	638459244	625952005	-
Fayoumi injection replicate 1	A	197411944	172932863	168938685	85.6%
	B	159555026	140727533	137559756	86.2%
Fayoumi injection replicate2	A	199785024	183003082	178916526	89.6%
	B	238290540	214461486	209933966	88.1%
	All	795042534	711124964	695348933	-
Leghorn control	A	200045434	185242072	181192144	90.6%

replicate 1					
	B	156119182	151930596	148781580	95.3%
Leghorn control replicate 2	A	293866348	264479713	261247183	88.9%
	B	516069410	447432178	437103730	84.7%
	All	1166100374	1049084559	852468090	-
Leghorn injection replicate 1	A	154123370	136245059	130080124	86.4%
	B	275142582	226717488	221214636	80.4%
Leghorn injection replicate 2	A	230330448	215619084	210982690	91.6%
	B	166882728	153031462	149740592	89.7%
	All	826479128	731613093	712018042	-

The chicken reference genome GRCg6a was downloaded from the Ensemble database.

Table S3. Differentially methylated sites and regions among groups.

**Class**	**FA *vs.* FP**	**LA *vs.* LP**
DMSs	2143	162
DMRs	102	10

The significance level of DMSs was set as FDR-corrected P-value below 0.01. DMRs were obtained by neighboring differentially methylated sites with P-value below 0.01.

Table S4. Pathway annotation of genes located within differentially methylated regions (DMRs) in FA compared to FP and LA compared to LP.

	GO.ID	Term	P
Fayoumi	GO:0030098	lymphocyte differentiation	0.01100
	GO:0009581	detection of external stimulus	0.01452
	GO:0045321	leukocyte activation	0.01542
	GO:0002313	mature B cell differentiation involved	0.02537
	GO:0006953	acute-phase response	0.02537
	GO:0033151	V(D)J recombination	0.03033
	GO:0002521	leukocyte differentiation	0.03217
	GO:0002366	leukocyte activation involved in immune response	0.03255

	GO:0002335	mature B cell differentiation	0.03282
	GO:0002263	cell activation involved in immune response	0.03363
	GO:0002704	negative regulation of leukocyte mediated immunity	0.04024
	GO:0070670	response to interleukin-4 stimulus	0.04024
	GO:0071353	cellular response to interleukin-4	0.04024
	GO:0030217	T cell differentiation	0.04043
	GO:0050727	regulation of inflammatory response	0.04043
	GO:0051606	detection of stimulus	0.0422
	GO:0002886	regulation of myeloid leukocyte mediated immunity	0.04271
	GO:0043300	regulation of leukocyte degranulation	0.04516
	GO.ID	Term	P
Leghorn	GO:0050896	response to stimulus	0.00376
	GO:0045637	regulation of myeloid cell differentiation	0.0086
	GO:0002762	negative regulation of myeloid leukocyte	0.01254
	GO:0030851	granulocyte differentiation	0.01254
	GO:1903707	negative regulation of hemopoiesis	0.01308
	GO:0050853	B cell receptor signaling pathway	0.01803
	GO:1902106	negative regulation of leukocyte differentiation	0.04253
	GO:0045596	negative regulation of cell differentiation	0.00731
	GO:0008219	cell death	0.0077
	GO:0016265	death	0.0077
	GO:0008285	negative regulation of cell proliferation	0.01091
	GO:0006915	apoptotic process	0.02268
	GO:0012501	programmed cell death	0.02586
	GO:0045596	negative regulation of cell differentiation	0.00731

Fayoumi individuals with AIV inoculation (FA) *vs.* Fayoumi individuals challenged with PBS (FP) FA *vs.* FP ($P < 0.05$ adjusted by FDR) and Leghorn individuals with AIV inoculation (LA) *vs.* Leghorn individuals challenged with PBS (LP) ($P < 0.05$ adjusted by FDR).

Table S5. T-test comparison of gene methylation difference in immune response related genes for Fayoumi and Leghorn chickens.

Gene	*P* value	Mean1	Mean2	Difference	SE of difference	t ratio	df
TLR4	0.2582	0.0407	0.1561	-0.1154	0.0924	1.2488	6.0
TLR3	0.9112	0.0847	0.0742	0.0104	0.0898	0.1163	6.0
TLR7	0.4080	0.0544	0.1155	-0.0611	0.0687	0.8894	6.0
IRF7	0.1485	0.0317	0.2067	-0.1750	0.1056	1.6577	6.0
IFNA3	0.7522	0.1156	0.1675	-0.0519	0.1570	0.3305	6.0
JAK1	0.1478	0.0309	0.1290	-0.0981	0.0591	1.6610	6.0
JAK2	0.3293	0.4000	0.1450	0.2550	0.2402	1.0617	6.0
IRF9	0.7231	0.2594	0.3587	-0.0993	0.2673	0.3714	6.0
CIITA	0.5890	0.0493	0.0888	-0.0395	0.0692	0.5706	6.0
MHC_class_II_BLB1	0.2520	0.1404	0.4404	-0.2999	0.2367	1.2672	6.0
BLB1	0.3012	0.1493	0.3794	-0.2300	0.2034	1.1311	6.0
ENSGALT00000041159	0.5395	0.1161	0.1773	-0.0613	0.0942	0.6504	6.0
MX1	0.3591	0.1311	0.0462	0.0848	0.0854	0.9930	6.0
ADAR	0.4035	0.1382	0.3122	-0.1740	0.1936	0.8985	6.0
CASP1	0.3765	0.0768	0.1751	-0.0984	0.1030	0.9549	6.0
IL1B	0.3307	0.0358	0.1177	-0.0819	0.0774	1.0582	6.0
IL18	0.1349	0.0381	0.1534	-0.1153	0.0668	1.7271	6.0
TNFAIP8L3	0.4126	0.0849	0.1876	-0.1027	0.1166	0.8803	6.0
NOS2	0.2740	0.0587	0.1599	-0.1012	0.0840	1.2037	6.0
CD4	0.1069	-0.0175	0.0950	-0.1124	0.0593	1.8950	6.0
SASH3	0.3698	0.0925	0.2463	-0.1538	0.1587	0.9693	6.0
ACOT8	0.2873	0.1303	0.4073	-0.2770	0.2373	1.1675	6.0
CD8A	0.9121	0.1037	0.1130	-0.0093	0.0808	0.1151	6.0
GPR18	0.4370	0.0359	0.0869	-0.0510	0.0612	0.8325	6.0
JCHAIN	0.4891	0.0509	0.1094	-0.0586	0.0795	0.7367	6.0
THOC1	0.7467	0.0696	0.0959	-0.0263	0.0778	0.3384	6.0

HSP90AB 1	0.3520	0.0584	0.1785	-0.1201	0.1190	1.0090	6.0
HSP90B1	0.9189	0.0930	0.1087	-0.0157	0.1480	0.1061	6.0
IL4I1	0.4600	0.0538	0.1691	-0.1153	0.1460	0.7893	6.0
IL13	0.6766	0.0820	0.1240	-0.0420	0.0958	0.4381	6.0
IL13RA1	0.3708	0.0444	0.1057	-0.0613	0.0634	0.9671	6.0
IL13RA2	0.4246	0.0705	0.1505	-0.0799	0.0934	0.8564	6.0
IL2RB	0.8964	0.0929	0.0782	0.0147	0.1078	0.1359	6.0
CASP9	0.4439	0.1064	0.2348	-0.1284	0.1567	0.8194	6.0
NOX2	0.2858	0.0347	0.1042	-0.0695	0.0593	1.1715	6.0
COQ10B	0.9162	0.0871	0.0798	0.0072	0.0659	0.1097	6.0
NCOA4	0.5146	0.0720	0.1184	-0.0464	0.0670	0.6924	6.0
FERMT1	0.4697	0.0438	0.0978	-0.0540	0.0699	0.7716	6.0
FERMT2	0.4347	0.0756	0.1339	-0.0583	0.0696	0.8370	6.0
ATG5	0.5013	0.0533	0.1027	-0.0494	0.0691	0.7153	6.0

"Mean1" represents the mean of gene methylation change degrees in immune response related genes for Fayoumi; "Mean2" represents the mean of gene methylation change degrees in immune response related genes for Leghorn; "Difference" represents the difference between "Mean1" and "Mean2"; "SE" represents the standard error of mean.

Table S6. Fixation index (F_{ST}) statistics summary for each chromosome.

Chromosome	Mean	Median	Variance
1	0.7726489	0.9197408	0.09789758
2	0.7543121	0.9195518	0.1095103
3	0.7862264	0.9310343	0.0971366
4	0.7657345	0.9267896	0.104616
5	0.7581064	0.9227512	0.1020113
6	0.5276487	0.6135947	0.0896215
7	0.5346708	0.6223336	0.09845613
8	0.5126336	0.6136161	0.1016923
9	0.5186245	0.6041661	0.09145366
10	0.8419605	0.9467218	0.07090918
11	0.8132281	0.9389565	0.0864009
12	0.7864329	0.9393952	0.106014

13	0.8170616	0.9457804	0.08102118
14	0.8444635	0.9512922	0.06645462
15	0.8490079	0.9520578	0.06988065
16	0.684984	0.6973741	0.04061274
17	0.8440361	0.9481538	0.07249616
18	0.8202319	0.950477	0.08640785
19	0.8260616	0.952555	0.08757863
20	0.8400858	0.9508067	0.07455204
21	0.9063933	0.955118	0.02464836
22	0.8609215	0.960644	0.04903138
23	0.8483695	0.9454929	0.06044839
24	0.8520206	0.9432012	0.05600844
25	0.7289529	0.9011074	0.1045757
26	0.864155	0.9471116	0.04983876
27	0.8317456	0.9329393	0.04778067
28	0.7563782	0.8930448	0.08109866
All	0.7470665	0.9137907	0.1043984
